# Genome of Rhizobium sp. UR51a, Isolated from Rice Cropped in Southern Brazilian Fields

**DOI:** 10.1128/genomeA.00249-15

**Published:** 2015-04-02

**Authors:** Rocheli de Souza, Fernando Hayashi Sant’Anna, Adriana Ambrosini, Michele Tadra-Sfeir, Helisson Faoro, Fabio Oliveira Pedrosa, Emanuel Maltempi Souza, Luciane M. P. Passaglia

**Affiliations:** a Departamento de Genética, Instituto de Biociências, Universidade Federal do Rio Grande do Sul (UFRGS), Porto Alegre, RS, Brazil; b Departamento de Bioquímica e Biologia Molecular, Universidade Federal do Paraná (UFPR), Centro Politécnico, Curitiba, PR, Brazil

## Abstract

Rhizobium sp. UR51a is a Gram-negative bacterium isolated from roots of rice plants, and it presents plant growth-promoting abilities. The nutrient uptake in rice plants inoculated with UR51a was satisfactory. The genome of strain UR51a is composed of 5,233,443-bp and harbors 5,079 coding sequences.

## GENOME ANNOUNCEMENT

Plant growth-promoting bacteria can enhance plant growth and nutrient uptake (1), and they are considered good candidates for crop inoculation. Bacteria of the Rhizobium genus are traditionally classified as symbiotic bacteria that fix atmospheric nitrogen within nodules of leguminous plants. Rhizobium organisms can also act as nonsymbiotic bacteria associated with nonleguminous species of great agricultural importance (2–6).

Rhizobium sp. UR51a was isolated from the roots of rice plants collected from Uruguaiana (29°45′18″S, 57°05′16″W), in the state of Rio Grande do Sul, Brazil (5). This strain possesses plant growth-promoting abilities, such as siderophore and indolic compound production and biological nitrogen fixation. Satisfactory results, obtained in relation to the nutrient uptake of rice plants inoculated with UR51a, indicated that this strain provides essential nutrients to plants and/or enables them to access more nutrients from the soil (R. de Souza, R. Schoenfeld and L. M. P. Passaglia, submitted for publication).

The genomic DNA of strain FeS53a was extracted and constructed into a 500- to 1,200-bp insert library. The whole-genome sequencing was performed with MiSeq Illumina platforms using the MiSeq regent kit v2. The assembly was initially tested using four software packages: CLC Genomics Workbench, A5-miseq (7), CISA (8), and SPAdes (9); the A5-miseq was chosen due to the lower value of *N*_50_ and fewer contigs. To assess the quality of the microbial genome, the CheckM program (10) was used with a completeness of 99.91%. Finally, the automatic annotation and classification of sequences were obtained using the RAST server (11).

The sequencing of UR51a resulted in 496,691 high-quality paired-end reads (approximately 39-fold coverage), with an average length of 250 bp. The 54 contigs obtained were connected to form 32 scaffolds, based on the paired-end relationships of the reads. The draft genome sequence of strain UR51a consisted of 5,233,443 bp, with a G+C content of approximately 59.25%. Fifty-one tRNA genes, 5 rRNA genes, and 5,079 coding sequences (CDSs) were assigned on the basis of the annotation.

The UR51a genome contains genes for the siderophore aerobactin uptake system (*fhuABCD*). Siderophores are involved in the sequestration of Fe^3+^ in soils with low iron content, and they can stimulate bacterial and plant growth by improving Fe^3+^ nutrition. Genes for auxin biosynthesis, an important regulator that can influence plant development, were also encountered. The UR51a genome encodes antioxidant enzymes that mediate the protection from reactive oxygen species generated by the plant hosts, as well as genes involved in resistance to antibiotics and toxic compounds. Similarly, the sequencing of the UR51a genome will increase the understanding of interaction mechanisms between this bacterium and rice, evidencing the future application as a biotechnological product to enhance crop nutrition and production.

### Nucleotide sequence accession numbers.

This whole-genome shotgun project has been deposited at GenBank under the accession number JYFU00000000. The version described in this paper is version JYFU01000000.
